# Sex differences in the association of photoperiod with hippocampal subfield volumes in older adults: A cross‐sectional study in the UK Biobank cohort

**DOI:** 10.1002/brb3.1593

**Published:** 2020-04-28

**Authors:** Naif A. Majrashi, Trevor S. Ahearn, Justin H. G. Williams, Gordon D. Waiter

**Affiliations:** ^1^ Aberdeen Biomedical Imaging Centre University of Aberdeen Aberdeen UK; ^2^ Diagnostic Radiology Department College of Applied Medical Sciences Jazan University Jazan Saudi Arabia; ^3^ Institute of Medical Sciences University of Aberdeen Aberdeen UK

**Keywords:** brain plasticity, FreeSurfer, hippocampus, MRI, sex differences

## Abstract

**Introduction:**

Even though seasonal and sex‐dependent changes in hippocampal and subfield volumes are well known in animals, little is known about changes in humans. We hypothesized that changes in photoperiod would predict changes in hippocampal subfield volumes and that this association would be different between females and males.

**Methods:**

A total of 10,033 participants ranging in age from 45 to 79 years were scanned by MRI in a single location as part of the UK Biobank project. Hippocampal subfield volumes were obtained using automated processing and segmentation algorithms using the developmental version of the FreeSurfer v 6.0. Photoperiod was defined as the number of hours between sunrise and sunset on the day of scan.

**Results:**

Photoperiod correlated positively with total hippocampal volume and all subfield volumes across participants as well as in each sex individually, with females showing greater seasonal variation in a majority of left subfield volumes compared with males. ANCOVAs revealed significant differences in rate of change in only left subiculum, CA‐4, and GC‐ML‐DG between females and males. PLS showed highest loadings of hippocampal subfields in both females and males in GC‐ML‐DG, CA1, CA4, subiculum, and CA3 for left hemisphere and CA1, GC‐ML‐DG, CA4; subiculum and CA3 for right hemisphere in females; GC‐ML‐DG, CA1, subiculum, CA4 and CA3 for left hemisphere; CA1, GC‐ML‐DG, subiculum, CA4 and CA3 for right hemisphere in males.

**Conclusion:**

The influence of day length on hippocampal volume has implications for modeling age‐related decline in memory in older adults, and sex differences suggest an important role for hormones in these effects.

## Graphical abstract

Photoperiod correlated positively with total hippocampal volume and all subfield volumes in all participants as well as in each sex individually, with females showing greater seasonal variation in a majority of left subfield volumes compared with males. There were significant differences in rates of change (slopes) between the subfields in both hemispheres between sexes. ANCOVAs revealed significant differences in rate of change in only left subiculum, CA‐4 and GC‐ML‐DG between females and males. PLS showed highest loadings of hippocampal subfields in both females and males in GC‐ML‐DG, CA1, CA4, subiculum, and CA3 for left hemisphere and CA1, GC‐ML‐DG, CA4, subiculum, and CA3 for right hemisphere in females; and GC‐ML‐DG, CA1, subiculum, CA4, and CA3 for left hemisphere and CA1, GC‐ML‐DG, subiculum, CA4, and CA3 for right hemisphere in males. The influence of day length on hippocampal volume has implications for modeling age‐related decline in memory in older adults, and sex differences suggest an important role for hormones in these effects.

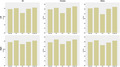

## 1 INTRODUCTION

Changes in environmental factors such as photoperiod (day length) play a major role in determining the behavior of most animals, particularly those further from the equator, affecting breeding, migration, and feeding pattern (Hut & Beersma, 2011). Changes in behavior are mediated by photoperiod in many mammalian species (Walton, Weil, & Nelson, 2011), and several studies have demonstrated that changes in behavior are accompanied by changes in brain volume or changes in specific regions. In particular, the volume of the hippocampus may be associated with photoperiod and be smaller in winter compared with summer (Clayton, Reboreda, & Kacelnik, 1997; Pyter, Reader, & Nelson, 2005; Workman, Manny, Walton, & Nelson, 2011; Yaskin, 2011). For example, white‐footed mice *Peromyscus leucopus* exhibit smaller hippocampal volume when exposed to shorter photoperiods (8‐hr day length) compared with those exposed to longer photoperiods (16‐hr day length), (Sherry & Hoshooley, 2010; Yaskin, 2011) and hippocampal mass is significantly decreased in bank voles during the winter season compared with the autumn or summer seasons (Yaskin, 2011). Furthermore, in rodents, deficits in behaviors such as spatial learning and memory that require an intact hippocampus have been reported when those rats are exposed to shorter day lengths compared with longer day lengths (Workman & Nelson, 2011; Yaskin, 2011). It has been proposed that changes in hippocampal volume and mass may result from a reduction in dendritic spine density in the CA1 and CA3 fields during the short days (Miller et al., 2015; Pyter et al., 2005), or that during long photoperiods there is increased dendritic branching complexity in the CA1 region. Additionally, reduced hippocampal mass in bank voles *Clethrionomys glareolus* in winter could be due to smaller dentate gyrus (DG), CA3, and CA4 hippocampal subfields compared with the autumn samples (Yaskin, 2013).

Sexual dimorphism in the structure of the nervous system is widely studied by the morphology of brain structures and development though not necessarily relating differences in behavior. The hippocampus is considered the most important brain region responsible for spatial information about the environment (Redish & Touretzky, 1998). It has been shown that sexual dimorphism in hippocampal size occurs in rodents and several bird species, and in all cases, hippocampal size is greater in animals of the sex group characterized by higher spatial activity (Redish & Touretzky, 1998; Yaskin, 2013). Seasonal changes in the environment (photoperiod) have been found to modulate sex‐related differences in hippocampal volumes and spatial activity (Yaskin, 2013). In particular, male bank voles *C. glareolus* exhibited higher hippocampal growth (19%–28%) which coincided with an increase in spatial activity (foraging behavior) in spring compared with females (8%–20% of growth), while in winter hippocampal sizes decreased and coincided with lower spatial activity (reduction in home ranges) and did not differ significantly between males and females. Furthermore, as well as total volume, hippocampal subfield volumes vary significantly according to sex and season in wild rodents (Burger, Saucier, Saucier, & Iwaniuk, 2013). In particular, dentate gyrus and CA‐3 found to be significantly larger in male compared with females in nonbreeding season. All together, these results suggest that seasonal sex‐related differences in behavior alter the hippocampal sizes differentially according to sex. It has been suggested that this is because changes in spatial memory are required as adaptations to greater territorial and ranging behavior in autumn and summer months, and that these differ according to sex.

Seasonal changes in hippocampal volume in humans have been demonstrated by Miller et al., but it was not of sufficient magnitude to determine whether specific areas were affected or whether changes were sex‐dependent. In this study, we hypothesize that seasonal changes are associated with not only total hippocampal volume but also with hippocampal subfield volumes. We also hypothesize that this association would be sex‐dependent and that females would be more sensitive to seasonal changes than males. We predicted that participants scanned on days with a long photoperiod would have larger hippocampal and subfield volumes compared to those scanned on days with a short photoperiod.

## 2 METHODS

### 2.1 Participants

From 2006 and 2010, 502,655 participants aged 37–73 years were recruited to the UK Biobank cohort. Participants attended one of 22 assessment centers across the UK and completed a range of lifestyle, demographic, health and mood questionnaires, cognitive assessments and physical measures (Allen et al., 2012; Sudlow et al., 2015), and subsequently brain imaging at a single center between 2014 and 2016 (Miller et al., 2016). The 10,103 participants aged between 45 and 79 years (mean = 62.5, *SD* = 7.4) in the January 2017 brain imaging data release were included in this cross‐sectional study. Seventy individuals in total were excluded from the study; six were excluded because of failure of the segmentation algorithm (the segmentation was aborted for more than three times), and sixty‐four were excluded due to issues related to the quality of their original T1 structural images (poor contrast and low signal to noise ratio (SNR)) before the processing. All segmentations were visually inspected. No other exclusion criteria were applied. All UK Biobank participants gave written, informed consent. UK Biobank received ethical approval from the North West Multi‐Centre Research Ethics Committee (11/NW/03820). This research was conducted using the UK Biobank Resource under Application Number 24089 (PI Waiter). All UK Biobank methods were performed in accordance with the UK regulations (https://www.ukbiobank.ac.uk/gdpr/).

### 2.2 Environmental variable (photoperiod)

Photoperiod in hours of daylight on the day of scan was derived from the latitude and longitude of the scanning center using the United States Naval observatory online data repository (http://aa.usno.navy.mil/data/docs/RS_OneYear.php). Photoperiod in hours was calculated by subtracting sunset from sunrise on the day of scan.

### 2.3 MRI acquisition

MRI scans were acquired using a 3T Siemens Skyra with a standard Siemens 32‐channel RF receive head coil (Miller et al., 2016). T1‐weighted 3D magnetization‐prepared rapid gradient echo (MPRAGE) images were acquired in the sagittal plane within 5 min with these parameters: resolution 1 × 1 × 1 mm, TR = 2,000 ms, TI = 880 ms, field‐of‐view 208 × 256 × 256 mm, iPAT = 2, and superior inferior field‐of‐view 256 mm (Miller et al., 2016).

### 2.4 Volumetric segmentation and analysis

Volumetric processing and segmentation were performed using the developmental version of the FreeSurfer v 6.0 software package (http://surfer.nmr.mgh.harvard.edu), with hippocampal subfield segmentation (Iglesias et al., 2015). We chose FreeSurfer (developmental version) because it is unbiased and has high accuracy and longitudinal reproducibility in hippocampal and subfield segmentations (Iglesias et al., 2015; Marizzoni et al., 2015; Van Leemput et al., 2009; Van Leemput et al., 2009) compared with the previous methods.

FreeSurfer was used to process the data including transformation to Talairach image space, nonuniform intensity normalization for intensity inhomogeneity correction, removal of nonbrain tissues using hybrid watershed, and segmentation of subcortical volumetric structures; white matter and deep gray matter (Fischl, 2012; Ségonne et al., 2004). For segmentation of the hippocampal subfields, the algorithm is based on combining manual labels from ex vivo (15 autopsy samples scanned at ultrahigh‐resolution (0.13 mm)) and in vivo T1 MRI scans of the whole brain (1‐mm resolution) to establish an atlas of the hippocampal formation with a new Bayesian inference algorithm to detect local variations in MRI contrast (Iglesias et al., 2015). For each subject, volumetric data for these subcortical volumes were calculated using the software's automatic Bayesian segmentation technique (Iglesias et al., 2015). Intracranial volume (ICV) and total brain volume (white matter plus gray matter) were also calculated by FreeSurfer using the Talairach transformation matrix created from the registration of normalization and MNI atlas (Buckner et al., 2004).

**Figure 1:**
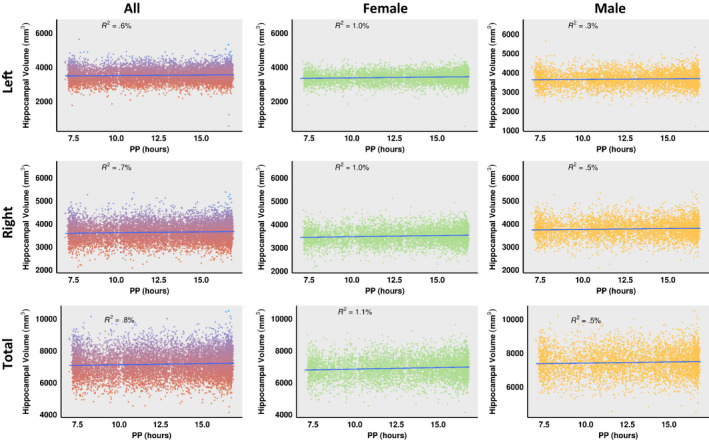
(a) Segmentation of hippocampal subfields by FreeSurfer in sagittal (top) and axial (bottom) images. (b) Schematic of coronal section showing the anatomy of the subfields of hippocampal formation: DG; dentate gyrus and CA; cornu ammonis. (c) Schematic of coronal section showing rate of change in left and right hippocampal subfield volumes per unit hour (mm^3^/hr.) for females and males

The FreeSurfer algorithm results in the segmentation of twelve distinct hippocampal subfields per hemisphere and these include: the dentate gyrus, CA1, CA2/3, CA4, fimbria, hippocampal‐amygdaloid transition area (HATA), hippocampal tail, molecular layer, parasubiculum, presubiculum, subiculum, and hippocampal fissure. We included the following subfields in our study: the granule cell and molecular layer of the dentate gyrus (GC‐ML‐DG), cornu ammonis: CA‐1, CA‐3 (noted as CA3 due to the indistinguishable MR contrast between CA2 and CA3) and CA‐4, and subiculum (Figure 1), and excluded the parahippocampal gyrus (presubiculum and parasubiculum), HATA, molecular layer, hippocampal tail and fissure and fimbria from our study because we were interested in only the subfields of the hippocampal formation. All hippocampal subfield volumes scale with whole head size; therefore, all volumes were corrected for total brain volume (TBV), age, and gender (O'Brien et al., 2011). Every image was visually inspected for segmentation quality by NAM. No manual interventions were performed on the data. Volumetric data were extracted from FreeSurfer and used for statistical analyses.

### 2.5 Statistical analysis

Statistical analyses were conducted with SPSS version 24. The alpha significance levels were Bonferroni‐corrected for (a) multiple regression analyses (for analyzing total hippocampal volume across all participants) and set at *p* (.05/3) = .016, and (b) for repeated multiple regression analyses among sexes and ANCOVA (when analyzing hippocampal subfield volumes across sexes) and set at *p* (.05/20) = .0025. The *p*‐value reported throughout the paper was Bonferroni‐corrected for multiple comparisons to minimize the likelihood of type I (false positive) statistical errors.

For total hippocampal volume analyses, Pearson (bivariate) correlations between photoperiod and left, right, and total hippocampal volumes as well as age, and total brain volume (TBV) were performed across all participants and in each sex individually. To investigate the predictability for each of these independent variables for the total hippocampal volumes, single linear regression models for each were created. There were significant correlations between total hippocampal volumes and age and TBV; therefore, these predictors were included as covariates in a multiple regression model.

For hippocampal subfields, a series of multivariate (GLM) analyses were conducted for each hemisphere using all hippocampal subfield volumes (corrected for age and TBV) as the dependent variables; sex as a fixed factor; and photoperiod as an independent variable (covariate). When the initial model (a series of repeated measurement (GLM) regression model) was significant across all hippocampal subfield volumes, a univariate regression model to assess the correlation of photoperiod between hippocampal subfield volumes across all participants and between sexes was then performed. A series of ANCOVA analyses (interactions between photoperiod and sex) to assess the difference in rate of change in left, right, and total hippocampal subfield volumes between females and males were performed. A predictive partial least square (PLS) regression model was performed to demonstrate (a) the variance accounted for by all left and right hippocampal subfield volumes (corrected for age and TBV) in the first latent factor and (b) weights (representing the correlation of photoperiod with each hippocampal subfield volume) and loadings (representing the direction of the of the relationship between photoperiod and subfield volumes). The significance of the first latent factor accounting for all left and right hippocampal volumes was tested by permutation test (5,000 iterations) using structural equation modeling of PLS.

## 3 RESULTS

### 3.1 Participant characteristics

Ten thousand and thirty‐three participants (52.2% females, 47.7% males), taken from the UK Biobank cohort, ranging from 45 and 79 years (mean = 62.5, *SD* = 7.4) from the January 2017 data release were included in this study. Three‐dimensional T1‐weighted images were collected from all participants. The MRI scans were acquired between May 2014 and December 2016, and the date of scan was recorded for each participant. Participants lived in approximately equal proportions north and south of the scanner and a mean distance of 31.1 km north or south of it (range 0.4–289 km). Therefore, location of residence would have a negligible effect on photoperiod and we used the photoperiod at the scanner location for all participants. The range of observed photoperiod in the UK Biobank Coordinating Centre (1–2 Spectrum Way, Adswood, Stockport UK) is from 7.49 hr in winter to 17.01 hr in summer. Demographic characteristics are presented in Table 1 below.

**Table 1:** The characteristics of the UK Biobank participants

Variables	All participants Mean (*SD*)	Females Mean (*SD*)	Males Mean (*SD*)
Number of participants	10,033	5,242	4,791
Age (years)	62.53 (7.4)	61.82 (7.3)	63.2 (7.6)
Left HC subfields			
Dentate Gyrus (mm^3^)	300.53 (37.1)	289.94 (32.1)	312.11 (38.8)
CA‐1 (mm^3^)	659.33 (80.4)	633.79 (69.9)	687.28 (81.8)
CA‐2/3 (mm^3^)	223.08 (32.8)	213.60 (28.3)	233.45 (34.3)
CA‐4 (mm^3^)	259.22 (31.6)	249.75 (27.5)	269.58 (32.7)
Subiculum (mm^3^)	435.38 (53.1)	419.58 (47.2)	452.68 (53.6)
Right HC subfields			
Dentate Gyrus (mm^3^)	314.19 (38.2)	302.65 (33.4)	326.82 (39.1)
CA‐1 (mm^3^)	685.84 (83.4)	659.83 (73.4)	714.30 (84.4)
CA‐2/3 (mm^3^)	242.71 (34.3)	232.64 (30.2)	253.73 (35.1)
CA‐4 (mm^3^)	271.96 (32.8)	261.78 (29.1)	283.09 (33.2)
Subiculum (mm^3^)	433.78 (51.1)	418.11 (45.4)	450.93 (51.2)
Left HC volume (mm^3^)	3,526.82 (383.3)	3,400.97 (333.2)	3,664.51 (387.2)
Right HC volume (mm^3^)	3,632.63 (390.2)	3,500.56 (341.3)	3,777.14 (389.1)
Total HC volume (mm^3^)	7,159.45 (752.4)	6,901.53 (652.9)	7,441.65 (752.8)
WMV (cm^3^)	470.47 (57.2)	443.87 (45.6)	500.13 (54.1)
GMV (cm^3^)	627.16 (55.2)	600.26 (44.1)	656.58 (51.1)
TBV (cm^3^)	1,097.90 (106.97)	1,044.14 (83.7)	1,156.72 (98.3)

Abbreviations: CA, cornu ammonis; GMV, gray matter volume; HC, hippocampus; *SD*, standard deviation; TBV, total brain volume; WMV, white matter volume.

### 3.2 Association of photoperiod (PP) with total hippocampal volume

There were significant linear correlations between photoperiod and total hippocampal volume across all participants. Photoperiod was positively correlated with left whole hippocampal (*r*(10,033) = .053, *B* = 6.57 ± 1.2 mm^3^/hr), right whole hippocampal (*r*(10,033) = .059, *B* = 7.56 ± 1.2 mm^3^/hr), and total (left + right) hippocampal (*r*(10,033) = .058, *B* = 14.14 ± 2.4 mm^3^/hr) volumes, *p* < .001. When photoperiod was corrected for age and TBV, correlations in left whole hippocampal (*r*(10,033) = .078, *B* = 6.92±0.88 mm^3^/hr), right whole hippocampal (r(10,033) = .086, *B* = 7.83±0.90 mm^3^/hr), and total (left + right) hippocampal (*r*(10,033) = .087, *B* = 14.75 ± 1.6 mm^3^/hr) volumes remained significant, *p* < .001 (see Figure 1).

**Figure 2:**
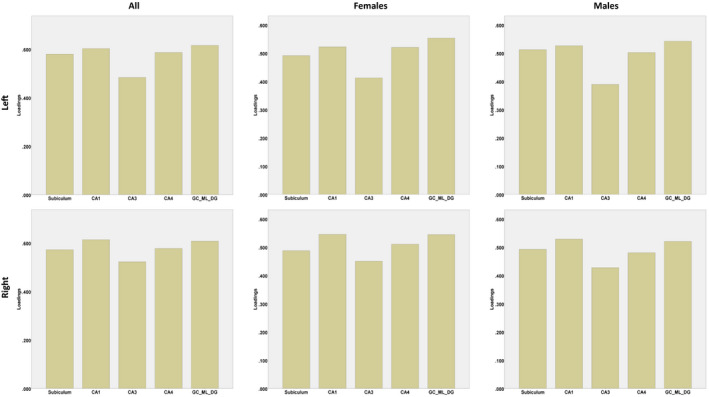
Linear correlations between photoperiod and left, right, and total hippocampal volumes in all participants, females, and males

### 3.3 Association of photoperiod with hippocampal subfield volumes

In the left hemisphere, the multivariate (GLM) regression model revealed a significant linear effect of photoperiod across all hippocampal subfield volumes (including all GC‐ML‐DG, CA1, CA2‐3, CA4, and subiculum) corrected for age and TBV (Wilk's Lambda = 0.993; *F* = 13.69; *df* = 5; Partial Eta squared = 0.007; Observed Power = 1.00; *p* = <0.001), and a significant effect of sex (Wilk's Lambda = 0.978; *F* = 44.68; *df* = 5; Partial Eta squared = 0.022; Observed Power = 1.00; *p* = <.001).

In the right hemisphere, the multivariate (GLM) regression model revealed a significant linear effect of photoperiod across all hippocampal subfield volumes (including GC‐ML‐DG, CA1, CA2‐3, CA4, and subiculum) corrected for age and TBV (Wilk's Lambda = 0.992; *F* = 16.13; *df* = 5; Partial Eta squared = 0.008; Observed Power = 1.00; *p* = <.001 in the right hemisphere), and a significant effect of sex (Wilk's Lambda = 0.992; *F* = 16.40; *df* = 5; Partial Eta squared = 0.008; Observed Power = 1.00; *p* = <.001).

The post hoc univariate regression analysis revealed a significant linear correlation of photoperiod between hippocampal subfield volumes in both hemispheres. Photoperiod was positively correlated with GC‐ML‐DG, CA1, CA2‐3, CA4, and subiculum volumes (corrected for age and TBV) in both hemispheres separately, *p* < .001 (see Table 2).

**Table 2:** Linear correlations between photoperiod and left, right, and total hippocampal subfield volumes in all participants, females, and males

	Subfield Volumes	All participants (*N* = 10,033)			Females (*N* = 5,242)			Males (*N* = 4,791)		
	*M* (*SE*)	*r*	*B* (*SE*)	*p*	*r*	*B* (*SE*)	*p*	*r*	*B* (*SE*)	*p*
Subiculum										
Left	435.3 (53.0)	.065	0.88 (0.13)	<.001	.089	1.1 (0.17)	<.001	.044	0.61 (0.20)	.003
Right	433.7 (51.0)	.068	0.89 (0.13)	<.001	.083	1.0 (0.17)	<.001	.055	0.76 (0.19)	<.001
CA‐1										
Left	659.3 (80.4)	.057	1.1 (0.19)	<.001	.081	1.5 (0.25)	<.001	.037	0.79 (0.30)	.011
Right	685.8 (83.4)	.063	1.2 (0.20)	<.001	.077	1.4 (0.26)	<.001	.050	1.1 (0.31)	.001
CA‐2/3										
Left	223.0 (32.8)	.061	0.55 (0.09)	<.001	.080	0.65 (0.11)	<.001	.049	0.48 (0.14)	.001
Right	242.7 (34.3)	.062	0.57 (0.09)	<.001	.066	0.56 (0.11)	<.001	.061	0.60 (0.14)	<.001
CA‐4										
Left	259.2 (31.6)	.074	0.58 (0.08)	<.001	.097	0.71 (0.10)	<.001	.053	0.45 (0.12)	<.001
Right	271.9 (32.8)	.077	0.64 (0.08)	<.001	.085	0.66 (0.10)	<.001	.071	0.63 (0.13)	<.001
GC‐ML‐DG										
Left	300.5 (37.1)	.075	0.68 (0.09)	<.001	.100	0.83 (0.11)	<.001	.053	0.52 (0.14)	<.001
Right	314.1 (38.2)	.082	0.78 (0.09)	<.001	.093	0.81 (0.12)	<.001	.072	0.74 (0.14)	<.001
Total HC	7,159.4 (752.4)	.087	14.8 (1.6)	<.001	.106	17.0 (2.2)	<.001	.070	12.6 (2.51)	<.001

Abbreviations: B, regression coefficient (mm^3^/hr); CA, cornu ammonis; GC‐ML‐DG, granulate cell of the molecular layer of the dentate gyrus; HC, hippocampus; M, volume mean; N, sample number; *p*, significance of *p*‐value (*p* < .05); *SE*, standard error.

A predictive PLS regression model revealed significant latent factors (via permutation test = 5,000 iterations) accounting for all left and right hippocampal subfield volumes separately (*p* < .001). The first latent factor accounted for 37.5% of the variance in the relationship between volume and photoperiod in both left and right hippocampal subfield volumes. Weights for each hippocampal subfield volume from the first latent factor are reported in Table 3. PLS showed highest loadings of hippocampal subfield volumes in; GC‐ML‐DG, CA1, CA4, subiculum, and CA3 for left hemisphere and CA1, GC‐ML‐DG, CA4, subiculum, and CA3 for right hemisphere (see Figure 2).

**Table 3:** Weights obtained from the first latent factor for each left and right hippocampal subfield volumes (corrected for age and TBV) across all participants, females, and males

Variables	Weights
All participants	
Left Subiculum	0.579
Left CA‐1	0.602
Left CA‐2/3	0.483
Left CA‐4	0.586
Left GC‐ML‐DG	0.615
Right Subiculum	0.572
Right CA‐1	0.613
Right CA‐2/3	0.522
Right CA‐4	0.577
Right GC‐ML‐DG	0.607
Females	
Left Subiculum	0.491
Left CA‐1	0.522
Left CA‐2/3	0.412
Left CA‐4	0.520
Left GC‐ML‐DG	0.553
Right Subiculum	0.487
Right CA‐1	0.545
Right CA‐2/3	0.450
Right CA‐4	0.510
Right GC‐ML‐DG	0.544
Males	
Left Subiculum	0.512
Left CA‐1	0.526
Left CA‐2/3	0.389
Left CA‐4	0.502
Left GC‐ML‐DG	0.542
Right Subiculum	0.492
Right CA‐1	0.528
Right CA‐2/3	0.427
Right CA‐4	0.480
Right GC‐ML‐DG	0.520

Abbreviations: CA, cornu ammonis; GC‐ML‐DG, granulate cell of the molecular layer of the dentate gyrus.

**Figure 3:**
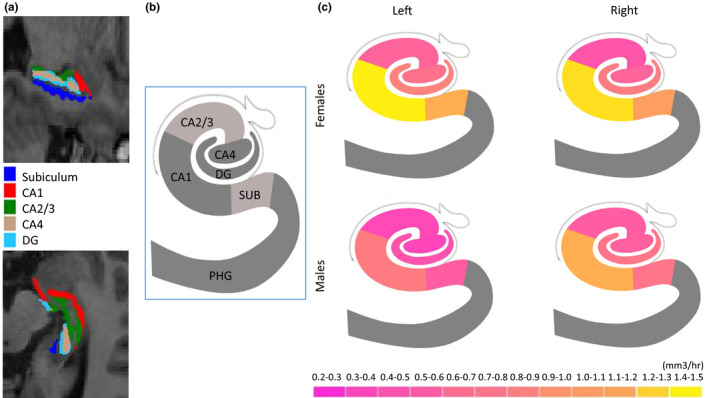
Loadings of left and right hippocampal subfield volumes (corrected for age and TBV) on the first latent variable (LV)

### 3.4 Sex differences in the association (rate of change) of total hippocampal and subfield volumes with photoperiod

For total hippocampal volume, there were significant linear correlations between photoperiod and total hippocampal volumes (corrected for age and TBV) in both females and males separately. Photoperiod was positively correlated with left whole hippocampal (*r*(5,242) = .099, *B* = 8.34 ± 1.1 mm^3^/hr), right whole hippocampal (*r*(5,242) = .101, *B* = 8.67 ± 1.1 mm^3^/hr), and total (left + right) hippocampal (*r*(5,242) = .106, B = 17.01 ± 2.2 mm^3^/hr) volumes in females as well as in males; and left whole hippocampal (*r*(4,791) = .059, *B* = 5.55 ± 1.3 mm^3^/hr), right whole hippocampal (*r*(4,791) = .074, B = 7.06 ± 1.3 mm^3^/hr), and total (left and right) hippocampal (*r*(4,791) = .070, *B* = 12.61 ± 2.5 mm^3^/hr) volumes, *p* <.001 (see Figure 2). The ANCOVAs revealed no significant interactions between sex and photoperiod on left whole hippocampal or right whole hippocampal or total (left + right) hippocampal volumes, *p* > .05. To sum up, there were no significant differences in rate of change in total hippocampal volumes accounted for photoperiod between sexes.

For left and right hippocampal subfield volumes, despite the multivariate analysis revealed a significant linear effect of sex across all hippocampal subfield volumes (corrected for photoperiod, age, and TBV) for both hemispheres (results described above), the post hoc univariate regression analysis revealed a significant linear effect of photoperiod between hippocampal subfield volumes in both hemispheres in both females and males separately (see Table 2). Photoperiod was positively correlated with GC‐ML‐DG, CA1, CA2‐3, CA4, and subiculum (corrected for age and TBV) in females and in males, *p* < .05, and that females showing greater rate of change in most of the subfields compared to males (see Table 2 and Figure 3). The ANCOVAs revealed significant interactions between sex and photoperiod on only left subiculum (*F* = 4.08; Partial Eta squared = 0.0004; Observed Power = 0.524; *p* = .043), left CA‐4 (*F* = 4.26; Partial Eta squared = 0.0004; Observed Power = 0.542; *p* = .039), and left DG (*F* = 5.09; Partial Eta squared = 0.001; Observed Power = 0.617; *p* = .024). To sum up, there were significant differences in rate of change in only left hippocampal subfield volumes (subiculum, CA‐44, DG), volumes accounted for photoperiod between sexes but not in the right hippocampal subfield volumes.

A predictive PLS regression model revealed significant latent factors (via permutation test = 5,000 iterations) accounting for all left and right hippocampal subfield volumes in both females and males separately (*p* < .001). The first latent factor in females accounted for 30.4% and 30.7% of variances in the relationship between volume and photoperiod in the left and right hippocampal subfield volumes, respectively, and the first latent factor in males accounted for 31.3% and 30.1% of variances in the relationship between volume and photoperiod in the left and right hippocampal subfield volumes, respectively. Weights for each hippocampal subfield volume from the first latent factor in both females and males separately are reported in Table 3. PLS showed highest loadings of hippocampal subfields in females and in males in; GC‐ML‐DG, CA1, CA4, subiculum, and CA3 for left hemisphere and CA1, GC‐ML‐DG, CA4, subiculum, and CA3 for right hemisphere in females; and GC‐ML‐DG, CA1, subiculum, CA4, and CA3 for left hemisphere and CA1, GC‐ML‐DG, subiculum, CA4, and CA3 for right hemisphere in males (see Figure 3).

## 4 DISCUSSION

In this study, we found a significant correlation between photoperiod and total and hippocampal subfield volumes within a large population cohort that survived correction for age and TBV. In addition, we found sex differences in the association (rate of change) of photoperiod with hippocampal subfield volumes with females showing greater rate of change compared with males and that sex differences were confined to the left side. Further, we found that GC‐ML‐DG and CA1 subfields in both hemispheres have the highest rate of change. Our findings that showed 0.7% of the variation in hippocampal volume was accounted for by variations in photoperiod on the day of scan consistent with a previous human study (Miller et al., 2015). In addition, our findings of the correlation between photoperiod with hippocampal subfield volumes are consistent with a number of previous animal studies (Pyter et al., 2005; Woolley, Gould, Frankfurt, & McEwen, 1990; Workman & Nelson, 2011), which have shown that hippocampal subfields, CA1 and CA3, were affected by changes in photoperiod.

To our knowledge, this study is the first to demonstrate sex‐related differences in the correlation of hippocampal subfield volumes with change of photoperiod in humans. The underlying biological mechanisms of sex‐related photoperiodic changes in hippocampal and subfield volumes are quite unclear. One possibility is that changes are mediated by effects of photoperiod on circadian rhythms mediated via the suprachiasmatic nucleus, a brain region that is considered the main circadian pacemaker (Tackenberg & McMahon, 2018). It has been shown that melatonin has direct effects on neurogenesis in the hippocampus but this would not explain sex differences in our study. It is known that adrenal cortical steroids and sex hormones control hippocampal neurogenesis (Yaskin, 2013; Zhang, Konkle, Zup, & McCarthy, 2008) and changing levels of androgens (for males) and estrogens (for females) have been shown to result in changes in hippocampal volume (Yaskin, 2013) with animal study showing that females have both larger hippocampal size and a high estradiol level in spring. However, the majority of females in our study would have been most‐menopausal and so systemic effects seem unlikely. However, the hippocampus synthesizes its own steroid hormones including estradiol and local levels may be orders of magnitude higher than systemic levels. Estradiol receptor levels are not known to be photoperiod sensitive but seasonal changes in brain Estradiol in the song sparrow have been demonstrated (Wacker, Wingfield, Davis, & Meddle, 2010). Estradiol induces phosphorylation of the CREB protein which is important in the formation of memory and may also have sex‐specific effects on spinogenesis.

Another important effect is through changes to circulating glucocorticoid levels that influence hippocampal volumes by modulating expression of brain‐derived neurotrophic factor (BDNF) (Binder & Scharfman, 2004; Sherman, Mumford, & Schnyer, 2015). Brain‐derived neurotrophic factor, which is a part of the family of nerve growth factor genes, has been shown to be an important protein that is responsible for control and survival of hippocampal neurons (Sheikhzadeh, Etemad, Khoshghadam, Asl, & Zare, 2015) and has been associated with nerve cell proliferation and hippocampal volume (Binder & Scharfman, 2004). Specifically, BDNF has been associated with high CA‐1 dendritic spine density. In summary, the relationships between hippocampal neurogenesis with the different sex hormones and adrenal cortical steroids are likely to be complex and are in need of further elucidation.

The changes we have found amount to approximately 15 mm^3^ per hour of daylight, from mid‐winter to mid‐summer, and we suggest that this amount in nontrivial. Normal age‐related atrophy in the elderly amounts to approximately 14 mm^3^ per year, and so the degree of seasonal variation relates to about 6 years of age‐related decline in an elderly population. This suggests that functioning in the elderly will be seasonally affected. Furthermore, studies are providing evidence for predominant Cornu Ammonis (CA‐1) and subiculum atrophy in MCI (Atienza et al., 2011) and AD (Frisoni et al., 2008; Wisse et al., 2014) suggesting that some parts of the hippocampus, and therefore, some mnemonic functions will be especially vulnerable. Furthermore, greater effects of the left side may also mean disproportionate effects on certain aspects of memory, given for example, that episodic or autobiographical memory is thought to be lateralized to the left side (Iglói et al., 2010).

Further longitudinal research will be required to determine whether some aspects of memory, especially in the elderly whose capacity is declining, are especially vulnerable to the short day–light hours of the winter, and whether this has an impact upon function. This cross‐sectional study included all data available in the January 2017 brain imaging data release. This means that we included participants who may suffer from depressive symptoms or may have medical or psychiatric issues related to their brain such as stroke, Alzheimer's disease, and congenital or acquired structural brain defects. In summary, our study provides compelling evidence that sexually influenced seasonal changes in the brain, already well recognized in many mammals and birds, extend to humans. Further research is required to determine the mechanisms underpinning these changes, and their functional importance, especially in elderly populations who may suffer particular deterioration in winter months.

## 5 CONCLUSION

Our study is the first to demonstrate the correlation of photoperiod with hippocampal subfield volumes within a large population cohort. In addition, our study is the first to demonstrate differences in the association of human hippocampal subfield volumes with photoperiod between sexes. We found that individuals scanned under long photoperiod conditions exhibited larger hippocampal volumes relative to those under short photoperiod conditions and that these effects are greater in females than males. These findings add to the evidence supporting the role of photoperiod on brain structural plasticity and could have implications for future investigations of human exposure to variations in natural light and artificial light and associated changes in mood.

## CONFLICT OF INTEREST

The authors declare that they have no potential conflict of interest and no competing interests.

## AUTHOR CONTRIBUTION

N.A.M. carried out the experiments, analyzed and interpreted the data, and wrote the manuscript. T.S.A. cosupervised the project and assisted with experimental interpretation and manuscript editing. J.H.G.W. codesigned the study and guided data interpretation and manuscript preparation. G.D.W. codesigned the study, cosupervised the project, and assisted with experimental interpretation and manuscript preparation.

## Data Availability

The datasets processed and analyzed during the current study are available from the online open access UK Biobank repository (https://www.ukbiobank.ac.uk/). This research was conducted under the UK Biobank Resource under Application Number 24089 (PI Waiter).
